# Transactions of the Epidemiological Society of London

**Published:** 1861-05

**Authors:** 


Art. XI.—*Transactions of the Epidemiological Society of London.* Vol. I., Part 1. 8vo., pp. 128. Richards: London, 1860.

The Epidemiological Society of London was founded, in 1850, for the purpose of investigating epidemic diseases, and of collecting and promulgating such facts, relating to the causes, origin, propagation, mitigation, prevention, and treatment of epidemic diseases, as appear to be established on sound and sufficient evidence, thus adding new views to the common stock of professional knowledge. On the outbreak of an epidemic in any place, it also purposes to institute a rigid examination into the causes of the first cases, and to ascertain whether, and by what means, they admit of prevention.

Abstracts of the monthly proceedings of the Society have appeared, from time to time, in the *Lancet, Medical Times and Gazette,* and *British Medical Journal;* but as these gave merely a general idea of the contents of many valuable papers, the council determined that the Society itself should publish its transactions. The present volume is, therefore, the first result of that determination, and it will be found to contain an interesting address by the president, Dr. Babington; a paper on the "Theory of Zymosis," by Dr. Richardson; a valuable report by the committee on "Diphtheria;" "Suggestions for Utilizing the Statistics of Disease among the Poor," by Dr. Milroy; and "Remarks on the Topography and Diseases of the Gold Coast," by Mr. Clarke.

